# Impact of Bisphenol A on the Cardiovascular System — Epidemiological and Experimental Evidence and Molecular Mechanisms

**DOI:** 10.3390/ijerph110808399

**Published:** 2014-08-15

**Authors:** Xiaoqian Gao, Hong-Sheng Wang

**Affiliations:** Department of Pharmacology, University of Cincinnati College of Medicine, Cincinnati, OH 45267, USA; E-Mail: gaoxq@mail.uc.edu

**Keywords:** BPA, cardiovascular, heart, mechanism

## Abstract

Bisphenol A (BPA) is a ubiquitous plasticizing agent used in the manufacturing of polycarbonate plastics and epoxy resins. There is well-documented and broad human exposure to BPA. The potential risk that BPA poses to the human health has attracted much attention from regulatory agencies and the general public, and has been extensively studied. An emerging and rapidly growing area in the study of BPA’s toxicity is its impact on the cardiovascular (CV) system. Recent epidemiological studies have shown that higher urinary BPA concentration in humans is associated with various types of CV diseases, including angina, hypertension, heart attack and coronary and peripheral arterial disease. Experimental studies have demonstrated that acute BPA exposure promotes the development of arrhythmias in female rodent hearts. Chronic exposure to BPA has been shown to result in cardiac remodeling, atherosclerosis, and altered blood pressure in rodents. The underlying mechanisms may involve alteration of cardiac Ca^2+^ handling, ion channel inhibition/activation, oxidative stress, and genome/transcriptome modifications. In this review, we discuss these recent findings that point to the potential CV toxicity of BPA, and highlight the knowledge gaps in this growing research area.

## 1. Introduction

Bisphenol A (BPA, CAS# 80-05-7), was first synthesized by Russian chemist Aleksandr Dianin in 1891 [1]. Since the 1950s BPA has been used in the manufacturing of polycarbonate plastics and epoxy resins. BPA-based products are tough, versatile and water-resistant and are used in various consumer goods such as food containers, baby bottles, beverage and food can linings, as well as for industrial purposes such as water pipes. Industrial production of BPA worldwide exceeds 2.2 million metric tons per year [2]. 

With the wide usage of BPA-based products, BPA has become a common environmental chemical. The polymerization reaction of BPA is not complete, leaving some unbound monomer BPA molecules in the products. BPA can be released into food or beverage over time, especially under heat, acidic or basic environments [3,4,5]. BPA exposure also occurs in human through inhalation [6]. It is estimated that the industrial synthesis process alone releases up to 100 tons of BPA into the atmosphere every year [7]. Multiple human exposure assessment studies have shown that BPA is at detectable levels in over 90% of individuals examined in various populations. Mean/median urinary BPA concentrations in the low μg/L (or low nM) range have been reported in various human exposure assessments [8,9,10,11,12]. 

BPA is an endocrine disrupting chemical (EDC). As early as in the 1930s, BPA was found to have estrogenic activities [1]. Later its ability to bind estrogen receptors and interfere with endogenous estrogens was demonstrated in various *in vitro* cell lines [13,14,15,16]. BPA can also bind to androgen receptors, exhibiting anti-androgenic effects *in vitro* [17]. Moreover, BPA is also shown to be able to bind to thyroid receptors, antagonizing thyroid hormones actions [18]. Other than the classic nuclear-receptor genomic signalings, BPA exerts some of its actions through non-genomic signaling without involving gene transcription processes [19]. Amplification of cell signaling enables very low dose ligand to impact final target proteins and alter cell functions in a rapid fashion. Rapid signaling of BPA has been reported in various systems, including breast cancer, pancreas, pituitary, cerebellar cortex and heart [20,21,22,23,24]. Extensive studies have suggested potential links between BPA exposure and diseases including cancer, obesity, diabetes and disorders of the reproductive, neuroendocrine and immune systems [25,26]. Recent evidence has also identified the CV system as a potential target of BPA. These findings suggest that BPA exposure may be a risk factor for a range of CV abnormalities such as vascular diseases and cardiac arrhythmias, and highlight the need for further assessment of the impact of BPA and other environmental EDCs on the CV system. Here, we provide a comprehensive review of current knowledge on BPA’s CV impact, with a focus on the underlying molecular mechanisms.

## 2. Impact of BPA on the CV System—Epidemiological Studies

So far the majority of epidemiological studies on the CV endpoints stems from the data set of the National Health and Nutrition Examination Survey (NHANES). In this survey, urine samples of participants were analyzed by Division of Environmental Health Laboratory Sciences using online solid-phase extraction coupled with high-performance liquid chromatography for total (free and conjugate) BPA concentration measurements. 

The first report of an association between higher urinary BPA level and CV diseases was from Lang *et al.* based on the 2003–2004 NHANES data [27]. The health effects of BPA in 1,455 adults were analyzed. It was found that weighted mean urinary BPA levels adjusted for age and sex were higher in those who were diagnosed with CV diseases including coronary artery heart disease, angina and heart attack. Statistically significant association were found between higher BPA concentrations and CV diseases (odds ratio, OR = 1.39 per 1-SD increase in BPA concentration) and diabetes, but not with other studied common diseases. Furthermore, when urinary BPA concentration values were divided into quartiles in fully adjusted logistic regression model, participants in the highest BPA concentration quartile had an OR of 2.89 for diagnoses of CV diseases comparing with lowest quartile participants. Subsequently, the same research group analyzed the data set from 2005 to 2006 NHANES [28]. It was shown that the geometric mean urinary BPA concentrations were lower in 2005–2006 NHANES than in the 2003–2004 cohorts (1.79 *vs.* 2.49 μg/L). After adjusting for confounding factors, it was found that in 2005–2006 NHANES, higher urinary BPA concentrations were still significantly associated with diagnosis of coronary artery disease (OR = 1.33 per 1-SD increase in BPA concentration), but not angina or myocardial infarction. It is likely that due to a 30% decrease in BPA concentrations in 2005–2006 NHANES participants, the power to detect associations with certain diagnoses of CV diseases was decreased. When data from 2003 to 2004 and 2005 to 2006 NHANES were pooled, all three diagnoses of cardiovascular diseases (coronary artery disease, angina and myocardial infarction) were significantly associated with higher BPA urine concentrations. 

Due to the cross-sectional nature of NHANES, it is possible that CV disease patients in the study altered their behaviors which resulted in increased BPA exposure and an apparent association between higher BPA and CV diseases. To strengthen their findings, in 2012, Melzer *et al.* analyzed a longitudinal study and assessed the effects of BPA on the development of coronary artery disease [29]. Included in the study were 861 controls and 758 incidents of coronary artery disease (CAD) followed for 10.8 years from the European Perspective Investigation of Cancer (EPIC)–Norfolk UK cohort. Using adjusted logistic regression model, it was demonstrated that higher urinary BPA concentrations were associated with incident CAD diagnosis (OR = 1.14 per 1-SD increase in BPA concentration). A later study by Melzer *et al.* published in 2012 addressed the possible mechanistic association between BPA exposure and CAD [30]. In this Metabonomics and Genomics in Coronary Artery Disease (MaGiCAD) study, patients underwent angiography carried out at Papworth Hospital NHS Trust, Cambridgeshire UK. It was found that higher urinary BPA was associated with angiography defined coronary atherosclerosis (OR = 2.09). It was suggest that coronary artery stenosis might be the causal link between high urinary BPA levels and CAD. In addition to CAD, higher BPA exposure has also been shown to be associated with peripheral arterial disease (PAD). PAD is known as a subclinical evaluation of vascular atherosclerosis and has strong association with development of CV diseases. In a study by Shankar *et al.*, PAD in 2003–2004 NHANES participants was defined according to American Diabetes Association 2003 guidelines, and an association between urinary BPA levels and incidence of PAD was found with a multi-variable adjusted OR of 1.38 [31]. 

The potential association between BPA exposure levels and CV disease risk factors has been examined. Shankar *et al.* studied 2003–2004 NHANES data and analyzed participants’ systolic and diastolic blood pressure [32]. As a major public health problem, hypertension is recognized as a significant risk factor for CV diseases. After adjusting for confounding factors, Shankar *et al.* found a positive association between higher levels of urinary BPA concentrations in participants and diagnoses of hypertension (OR = 1.50). The potential association between urinary BPA levels and heart rate variability was investigated in Korea elderly populations [33]. Heart rate variability (HRV) is a fine tuning mechanism of heart to adjust to constant changes in blood supply demand; decreased HRV is a risk factor for cardiac diseases. Consistent with the study by Shankar and Teppala [32], the study reported a positive association between higher urinary BPA concentrations and HRV as well as high blood pressure. 

It should be noted that a study by LaKind *et al.* pointed out that NHANES data may not be sufficient to draw the conclusion that BPA exposure was associated with CV diseases [34]. They noticed inconsistencies in previous NHANES studies in terms of methods and definitions, and applied consistent *a priori* chosen methods in their analysis of four NHANES data sets. Their results showed that urinary BPA was not associated with heart attack, coronary artery disease or diabetes in NHANES participants. The fact that different methodologies can result in inconsistent outcomes raised questions on how reliable the NHANES data is for establishing associations or cause-and-effect relationship between urinary BPA levels and CV diseases. It was suggested that conclusions should be made with caution using the NHANES surveys [35]. Further, in a cross-sectional study from the Prospective Investigation of the Vasculature in Uppsala Seniors (PIVUS), Olsen *et al.* found no connections between BPA levels and CV diseases risk factors [36]. Here, a Framingham Risk Score (FRS) system was used to predict the risk of CV diseases in elderly participants. The FRS system includes age, gender, history, blood pressure, blood lipid and glucose levels, and gives estimation of general CV disease risks. No association between serum BPA concentrations and FRS scores was identified. The usage of a broad CV risk estimate *versus* specific CV endpoints (e.g., CAD, hypertension) may contribute to the contrasting findings by Olsen *et al.* and other studies. 

In summary, multiple cross-sectional and longitudinal epidemiological studies have showed that in adults, BPA exposure levels are associated with CV diseases or CV disease risk factors. These include several independent analyses of NHANES demonstrating that participants’ reported CV diagnoses were associated with higher urinary BPA concentrations. No other diseases except diabetes reported by participants were associated with BPA urine levels in these studies, which may suggest a potential causal role of BPA in CV disease. On the other hand, no association between BPA exposure and CV disease was found in other studies, including one also using NHANES. Different methodologies used in the studies such as exclusion criteria, definitions and scoring systems may contribute to the inconsistency. The conflicting findings of the epidemiological results highlight the need for epidemiological studies with greater power and better design. 

## 3. Impact of BPA on the CV System—Experimental Studies

Various experimental studies have used animal models, *ex vivo* hearts, and *in vitro* cell systems to examine the CV impact and underlying mechanisms of BPA. These experimental studies can be divided into two categories: 1, “low-dose” BPA studies that focus on environmentally relevant doses, *i.e.*, common human exposure relevant doses and 2, studies using supra-physiological higher doses of BPA to identify its pharmacological and toxicological actions which may be relevant to specific poisoning conditions or occupational exposures. 

### 3.1. Definition of “Low-Dose” BPA in Experimental Studies

For EDCs, low-dose effects refer to the effects that occur in the range of human exposures or at doses lower than those used in traditional risk assessment toxicological studies [37,38]. Low-dose studies address environmental exposure levels of EDCs, and are more physiologically relevant to the impact of EDCs on the general population. For BPA, US National Toxicology Program (NTP) defined “low-dose” as doses lower than 50 mg/kg/day, which is the lowest observed adverse effect level (LOAEL) in traditional toxicological studies. Majority of *in vivo* laboratory animal studies use oral route to deliver BPA ≤ 50 mg/kg animal weight/day. For *in vitro* tissue and cell studies, “low-dose” cut-off was defined as ≤ 1 × 10^−7^ M by the Chapel Hill expert panel [19] and has been widely adopted. 

### 3.2. “Low-Dose” Experimental Studies—Rapid Impacts of BPA on the Heart

**Figure 1:**
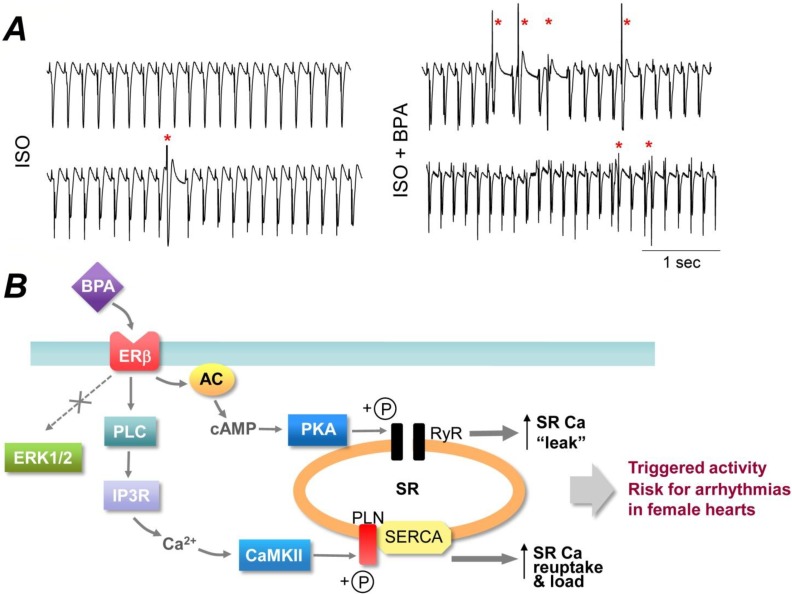
(**A**). Acute exposure to low-dose BPA promoted the development of ventricular arrhythmias under catecholamine-induced stress in female rat hearts. Shown are example surface ECG traces from female rat hearts under 10^−8^ M isoproterenol (ISO) alone or in the presence of 10^−9^ M BPA and ISO. Red asterisks indicate premature ventricular beats. Experimental methods are as described in [39]. Unpublished data by X Gao and H-S Wang. (**B**). Schematic illustration of the rapid signaling mechanism of BPA in female rat ventricular myocytes. Adopted from [24] with license permission.

Our laboratory investigated the acute effect of exposure to low-dose BPA on cardiac electrical rhythm in adult rat hearts [39]. Surface electrocardiogram (ECG) analysis of *ex vivo* rat hearts demonstrated that under catecholamine-induced stress condition, rapid exposure to 10^−9^ M BPA markedly increased the frequency of ectopic ventricular beats in a female-specific manner (Figure 1A). At the cardiac myocyte level, acute exposure (minutes) to 10^−9^ M BPA promoted the development of “triggered activities” in ventricular myocytes from female, but not male rat hearts. Triggered activities are aberrant spontaneous excitation of cardiac myocytes, and are recognized as one of the key mechanisms of cardiac arrhythmogenesis [40]. In another study, we examined the effect of acute BPA exposure on the development of cardiac arrhythmias following ischemia injury [41]. Ischemic reperfusion injury occurs during myocardial infarction (*i.e.*, heart attack), and can lead to irreversible cell damage, ventricular dysfunction, and ultimately heart failure. It also results in electrophysiological perturbation of the heart and acute arrhythmias, which can lead to sudden cardiac death [42,43]. It was shown that in female, but not male hearts, acute exposure to 10^−9^ M BPA during reperfusion resulted in a marked increase in the duration of sustained ventricular arrhythmias. These studies demonstrate that acute exposure to low-dose BPA has female-specific pro-arrhythmic actions in rodent hearts; the arrhythmogenic effect of BPA is particular pronounced under pathophysiological conditions such as stress or ischemic injury. 

These initial findings were followed by a series of studies to elucidate the underlying mechanisms of the rapid actions of BPA in the heart. We showed that BPA’s pro-arrhythmic effects in female rat cardiac cells were mediated at least in part by rapid alteration of myocyte Ca^2+^ handling [24,39]. Ca^2+^ handling is at the core of cardiac physiology, linking electrical excitation of the heart with mechanical contraction of the myocardium. Abnormalities in Ca^2+^ handling play a key role in cardiac arrhythmogenesis [40,44]. It was demonstrated that in female rat cardiac myocytes, 10^−9^ M BPA rapidly increased sarcoplasmic reticulum (SR) Ca^2+^ reuptake, Ca^2+^ release and SR Ca^2+^ load on a beat-to-beat basis. More importantly, diastolic spontaneous SR Ca^2+^ release, or SR Ca^2+^ “leak” was significantly increased by BPA treatment and suppression of aberrant SR Ca^2+^ release abolished the pro-arrhythmic effect of BPA. 

Of importance to the assessment of the potential toxicity of BPA and other EDCs are the non-monotonic dose responses of these chemicals [38,45]. A non-monotonic dose response curve is one that has a point of inflection where the curve slope switches sign from positive to negative or vice versa. We demonstrated that over the dose range of 10^−12^ to 10^−6^ M, the dose-responses of BPA in female rat ventricular myocytes were inverted-U shaped, or non-monotonic, as measured by multiple endpoints including myocyte mechanics, Ca^2+^ transient amplitude, and frequency of triggered activities [46]. The most efficacious dose of BPA’s cardiac actions was around 10^−9^ to 10^−8^ M, overlapping with the reported human exposure levels. Further, it was demonstrated that the non-monotonicity of BPA’s cardiac impact was produced by monotonic actions of BPA on individual myocyte Ca^2+^ handling processes. Over the examined dose range, BPA progressively increased SR Ca^2+^ release/leak and SR Ca^2+^ reuptake with monotonic dose responses, producing a stimulatory effect on myocyte mechanics and triggered activity development. Opposing this stimulatory effect, BPA progressively suppressed the L-type Ca^2+^ current, also with monotonic dose response; the resulting reduction in Ca^2+^ influx at high (micromolar) doses could account for the decline phase of the inverted-U shaped dose response. Thus, the non-monotonic dose response of BPA in rodent cardiac myocytes was produced by monotonic effects on multiple cellular Ca^2+^ handling processes. This represents a distinct mechanism of the non-monotonicity of BPA’s actions.

The mechanism underlying the gender specificity of BPA’s rapid cardiac effects involved the counterbalancing actions of estrogen receptor (ER) α and ERβ [24,39,47]. Using mechanical contraction as an index of myocyte Ca^2+^ cycling, we showed that activation of ERβ was stimulatory in both female and male rats, while activation of ERα was inhibitory in both genders. The responsiveness of cardiac myocytes to rapid BPA exposure was determined by the combined effects of ERα and ERβ signaling. Thus, the response of female cardiac myocytes to BPA was dominated by the stimulatory ERβ signaling, while in male cardiac myocytes the stimulatory effect of ERβ was counterbalanced and masked by the inhibitory ERα signaling, resulting in no observable response. The arrhythmogenic effects of BPA in female rodent cardiac myocytes were abolished by pharmacological blockade of ERβ and ablation of ERβ in knockout mice. Pharmacological blockade of ERα revealed the stimulatory effects of ERβ signaling in male myocytes, producing a female-like response to BPA in male cells. These results are the first to demonstrate that the opposing actions of ERs determine the sex specificity of BPA sensitivity.

The cardiac-specific molecular mechanism of BPA’s arrhythmogenic rapid action was elucidated in a recent study by our laboratory [24]. It was shown that in female rat cardiac myocytes, low-dose BPA exposure activated distinct signaling pathways including the Protein Kinase A (PKA) and Ca^2+^/CaM-dependent protein kinase II (CAMKII) signaling pathways. Acute BPA exposure resulted in increased production of cAMP, activation of PKA and phosphorylation of the ryanodine receptor (RyR); BPA also resulted in activation of phospholipase C (PLC), production of triphosphoinositol (IP_3_), IP_3_ receptor-mediated Ca^2+^ release, likely from the endoplasmic reticulum Ca^2+^ storage, and activation of CAMKII, which phosphorylated phospholamban (PLN). RyR phosphorylation led to enhanced channel opening and elevated SR Ca^2+^ leak, while PLN phosphorylation could release the inhibition of PLN on Sarco/Endoplasmic Reticulum Ca^2+^-ATPase (SERCA) and enhance SR Ca^2+^ reuptake. These two pathways were localized and only impacted their specific protein targets. Both pathways contributed to BPA-induced triggered activities and arrhythmogenesis in cardiac myocytes (Figure 1B). ERK1/2, which are commonly implicated in the rapid signaling cascade of BPA, were not involved in the cardiac effects of BPA in the heart. 

### 3.3. “Low-Dose” Experimental Studies—Effects of Chronic BPA Exposure

Several animal studies have been carried out to investigate the effects of long-term BPA exposure on the CV system. Patel *et al.* demonstrated BPA lifelong exposure altered cardiac function and structure in C57BL/6n mice in a sex-specific manner [48]. It was shown that low-dose BPA (5 μg/kg body weight/day) lifelong exposure induced cardiac concentric remodeling in male but not female mice, and increased systolic, diastolic and mean arterial pressures in female but not in male mice. Also, BPA exposure resulted in sex-specific alterations in the expression levels of Ca^2+^ handling proteins including SERCA, sodium-calcium exchanger (NCX), calsequestrin (CASQ2) and PLN, and in DNA methyltransferase expression and global DNA methylation in the heart. In males, SERCA2A, NCX1 and CASQ2 expressions were increased with BPA treatment, corresponding to increased intracellular Ca^2+^ removal and SR Ca^2+^ storage; while in females, the most significant change was increased PLN expression which likely led to reduced SR Ca^2+^ reuptake and Ca^2+^ load. These results were different from previous *in vitro* studies in isolated rat ventricular myocytes [24,39], likely reflecting the different impact of long-term and acute exposure and potential compensatory effects under long-term exposure.

Kim *et al.* showed that low-dose BPA (50 μg/kg body weight/day) accelerated atherosclerosis progress in a genetic mouse model prone to endothelial dysfunction and vascular inflammation [49]. An apolipoprotein E knockout (ApoE^−/^^−^) mice were fed BPA via drinking water for 12 weeks, which resulted in a significantly higher number of atherosclerosis lesions in the aorta compared with control. Non-HDL (non-high density lipoprotein) and total cholesterol levels in the mice were found to be correlated with aorta atherosclerosis lesion numbers. This finding is consistent with epidemiological human data and demonstrated an association between low-dose, chronic BPA exposure and atherosclerosis, although the underlying mechanisms are not yet understood. 

To investigate the underlying mechanisms of the potential BPA cardiotoxicity, Aboul Ezz *et al.* administered BPA orally to adult male rats for 6 to 10 weeks (25 or 10 mg/kg/day) and examined oxidative stress parameters in the heart [50]. They found increased lipid peroxidation, decreased reduced glutathione (GSH) level as well as decreased catalase activity, indicating that BPA exposure induced reactive oxygen species (ROS) production and compromised the function of mitochondria in the hearts of male rats. BPA exposure also resulted in a decrease in nitric oxide (NO) which may cause vasoconstriction and decreased blood supply to the heart. The activity of acetylcholinesterase was lower in the BPA-treated group, which may lead to reductions in heart rate and cardiac contraction. These results suggested that increased oxidative stress may play a role in the BPA’s potential adverse impact on the CV system. 

A recent work by Chapalamadugu *et al.* performed low-dose BPA exposure experiment on pregnant female rhesus monkeys, and investigated the impact of maternal BPA exposure on the CV system in the offspring [51]. Pregnant female rhesus monkeys were delivered 400 μg/kg body weight/day BPA either in early gestational or late gestational period. The fetal hearts were harvested after delivery and used for whole transcriptome analysis via microarray. It was found that maternal BPA exposure significantly altered fetal transcript expression profile in the fetal heart. They identified significant down-regulation of gene *Myh6* and up-regulation of *Adam12* in fetal ventricles, which were both shown to be related to CV pathology such as cardiac hypertrophy. This study provided evidences that maternal dietary exposure to BPA may have adverse impact on the offspring CV system. It was the first such study using primate as experimental subjects, and is of high relevance to humans. Follow-up studies are needed to determine the functional significance of the altered cardiac transcriptome profile, which would further elucidate the mechanistic link between BPA exposure and CV risks.

### 3.4. “High-Dose” Experimental Studies

Though not reflecting common environmentally-relevant exposure in the general population, these “high-dose” studies are of pharmacological interests and may provide understanding of BPA’s possible targets and signaling pathways involved in its CV toxicity. In addition, these studies may be of relevance to poison conditions, exposure under specific conditions, and occupational exposures. For instance, it is known that neonates in intensive care units, who have reduced metabolic capacities and are under constant exposure to medical devices, can have urinary BPA concentration in the micromolar range [52]. Because of chronic exposure to BPA containing products, 24 nM to 8.5 μM of BPA have been reported in urine samples from industrial workers [53]. Hospitalized patients who are under medical intervention and constant exposure to plastic medical tubing and equipment probably also have higher BPA concentration in their bodies [54]. 

Some studies that involved primarily high-dose but also examined low-dose are also discussed in this section. Using spontaneous beating atrial tissue isolated from rats, Pant *et al.* showed that BPA decreased atrial contraction rate and force at 10^−6^ to 10^−4^ M [55]. They showed that 10^−4^ M BPA depressed spontaneous atrial contraction force and rate by 90%, and these effects of BPA were mediated by NO-cGMP system. It was suggested that increased concentrations of NO or cGMP was likely the cause of depression of both pacemaker functioning and myocardial contractility. This result contrasts with that of Aboul Ezz *et al.* [50] showing decreased NO levels in rat hearts under low-dose BPA exposure, demonstrating the importance of BPA dose in experimental design. It has been shown that 17β-estradiol (E2) exhibited vasculoprotective effects in both animal models and *in vitro* cell systems, by attenuating migration and proliferation of vascular smooth muscle cells [56,57,58]. The effects of BPA on both female and male rat vascular smooth muscle cells were examined [59]. It was shown that 10^−5^ M BPA acted as an antagonist to E2’s inhibitory effects on migration of aorta vascular smooth muscle cells. The antagonism was likely mediated through ERβ receptors. 

Ion channels are fundamental for heart excitability and electrical conduction, and for physiological function of the vasculature. Several studies have shown that high-dose BPA affects the functioning of ion channels in the CV system. Asano *et al.* reported that at 10^−5^ to 10^−4^ M, BPA activated the large conductance Ca^2+^/voltage-sensitive K channels (Maxi-K) in canine and human coronary artery smooth muscle cells [60]. The activation by high-dose BPA of Maxi-K channels was dependent on β1 regulatory subunit and in a reversible, dose-dependent, non-genomic manner. O’Reilly *et al.* examined the effects of BPA on human heart sodium channels (hNa_v_1.5) transiently expressing in a heterologous cell system [61]. It was demonstrated that BPA blocked hNa_v_1.5 current with a K_d_ of 2.5 × 10^−5^ M. Also, the steady state inactivation of hNa_v_1.5 was shifted to more hyperpolarized potentials under 10^−4^ M BPA treatment, indicating its binding to inactivated state of the channels. The blockade of hNa_v_1.5 by BPA had similar properties as that by local anesthetics; the two share a common binding site on hNa_v_1.5 involving amino acid F1760. Later, Deutschmann *et al.* reported that BPA inhibited the L-type Ca^2+^ channels in isolated mouse ventricular myocytes in a dose-dependent manner with an IC_50_ of 2.5 × 10^−5^ M [62]. The blockade was fully reversible, and BPA likely exerted its action by binding to channels at the resting state. The binding site was probably located at the extracellular region of pore-forming subunit because no effects were observed when BPA was applied intracellularly. This study also tested a variety of other voltage-gated Ca^2+^ channels in native cells, and found that the N-, P/Q-, and T-type Ca^2+^ channels were inhibited by BPA with similar EC_50_ at μM range. A recent study demonstrated that BPA inhibited three subtypes of T-type Ca^2+^ channels expressed in HEK293 cells with different potency, and the inhibition showed different properties at low-dose (nM) and high-dose (μM) [63]. Low-dose BPA at nM range inhibited the channels with an order of potency Ca_v_3.2 ≥ Ca_v_3.1 > Ca_v_3.3 without affecting channel gating properties; while high-dose of BPA at μM accelerated current decay and shifted steady-state inactivation voltage dependence, and the inhibition potency was Ca_v_3.3 ≥ Ca_v_3.2 > Ca_v_3.1. 

A new study by Posnack *et al.* systematically investigated the rapid effects of BPA on electrophysiological function of *ex vivo* heart [54]. Excised adult female rat hearts were treated with BPA for 15 min and electrical activities of the hearts were recorded via both ECG electrodes and optical mapping. It was demonstrated that 10^−6^ to 10^−4^ M BPA prolonged ECG PR segment, decreased ventricular conduction velocity and increased action potential duration in *ex vivo* female rat hearts. At the highest dose of 10^−4^ M, BPA led to 3rd degree atrioventricular block. At low-dose (10^−7^ M), a modest but statistically significant reduction in ventricular conduction velocity was observed. Although direct evidence on the underlying mechanism of the observed electrical alteration was not provided, it was suggested that previously reported effects of BPA on cardiac sodium channels, Maxi-K channels, L-type Ca^2+^ channels and NO-cGMP signaling could all contribute to the observed dysfunction in myocardial electrical conduction. 

## 4. Conclusions

CV disease is a significant health problem worldwide and one of the major contributors to mortality and morbidity. The genetic, physiological and pathophysiological contributors to CV disease have been intensively studied; however, much less is known about the potential impact of environmental chemicals, including EDCs. Several epidemiological studies indicate that BPA exposure in adult populations is associated with increased risk for CV diseases, including coronary artery heart disease, angina, heart attack, hypertension, and peripheral artery disease. Experimental studies suggest that both acute and chronic BPA exposure at environmentally-relevant “low-dose” could affect the physiological functioning of CV system and promote abnormal CV activities such as arrhythmias, cardiac remodeling, atherosclerosis, and altered blood pressure. The underlying molecular mechanisms may involve estrogen receptor rapid signaling and alteration of cardiac Ca^2+^ handling through phosphorylation of Ca^2+^ handling proteins, alteration of cardiac Ca^2+^ handling protein expressions, ion channel inhibition/activation, oxidative stress, and genome/transcriptome modifications. Although the current evidence is still limited, it suggests that environmental exposure to BPA may be a contributing risk factor for CV disease, and points to the need for further assessment of the potential CV toxicity of BPA as well as other estrogenic EDCs. 

Significant gaps remain in our understanding of the CV impact of BPA. Future epidemiological studies should include additional CV endpoints such as arrhythmias and failure. Systematic and integrated experimental studies using animal models and *in vitro* systems are needed to further address the mechanistic link between BPA exposure, including acute, developmental and adult exposures, and abnormalities in specific CV endpoints. Knowledge on the cellular and molecular mechanisms of the biological/toxic impact of BPA is not only necessary for developing effective therapeutic treatment and protective measures, but also informative for epidemiological study design and methodologies by providing new markers and endpoints. While several epidemiological studies point to an association between BPA exposure and vascular diseases in humans, the impact of BPA on the vasculature as well as its underlying mechanism remain poorly understood and should be addressed. Further, BPA exposure alone at typical environmental levels may not result in clinically significant CV events in healthy individuals, but may be relevant in subpopulations with existing CV pathophysiological conditions. Consistent with this notion, it was shown that low-dose BPA did not result in detectable arrhythmia events in normal female rat hearts, but significantly exacerbated ventricular arrhythmias under stress condition or following ischemia injury [39,41]. Therefore, the impact of BPA exposure should be examined in the general population as well as in subpopulations with existing CV disease risk factors and susceptibility. This can also be addressed using CV disease animal models including large animals which share similar CV physiology with humans. 
